# Spontaneous Ultrasonic Vocalization Transmission in Adult, Male Long–Evans Rats Is Age-Dependent and Sensitive to EtOH Modulation

**DOI:** 10.3390/brainsci10110890

**Published:** 2020-11-22

**Authors:** Nitish Mittal, W. Todd Maddox, Timothy Schallert, Christine L. Duvauchelle

**Affiliations:** 1 Division of Pharmacology and Toxicology, College of Pharmacy, The University of Texas at Austin, 2409 University Avenue, Stop A1915, Austin, TX 78712, USA; niimits1@gmail.com; 2 Waggoner Center for Alcohol and Addiction Research, The University of Texas at Austin, 2500 Speedway, Stop A4800, Austin, TX 78712, USA; 3 Cognitive Design and Statistical Consulting, Austin, TX 78746, USA; wtoddmaddox@gmail.com; 4 Department of Psychology, Behavioral Neuroscience Division, The University of Texas at Austin, 108 E. Dean Keeton, Stop A8000, Austin, TX 78712, USA; schallert@psy.utexas.edu

**Keywords:** ultrasonic vocalizations, positive affect, negative affect, alcohol self-administration

## Abstract

Ultrasonic vocalizations (USVs) are well-established markers of motivational and emotional status. Recent work from our lab has provided novel evidence for a role of USVs in models of ethanol (EtOH) use. For instance, USV acoustic characteristics can be used to accurately discriminate between rats selectively bred for high EtOH intake (e.g., alcohol-preferring (P) and high-alcohol-drinking (HAD)) versus EtOH-avoiding (e.g., alcohol-non-preferring (NP) and low-alcohol-drinking (LAD)) strains, as well as differentiate between male and female rats. In the present study we sought to explore the effect of age and alcohol availability on spontaneously emitted 50–55 kHz frequency modulated (FM) and 22–28 kHz USVs in adult, male Long–Evans rats. With the hypothesis that age and alcohol experience influence spontaneous USV emissions, we examined USV data collected across a 24-week intermittent EtOH access experiment in male Long–Evans rats. USV counts and acoustic characteristic (i.e., mean frequency, duration, bandwidth and power) data revealed distinct age-dependent phenotypes in both 50–55 kHz FM and 22–28 kHz USV transmission patterns that were modulated by EtOH exposure. These results highlight the influence of age and EtOH experience on the unique emotional phenotypes of male Long–Evans rats.

## 1. Introduction

Researchers have long recognized that rats are a highly vocal species that emit ultrasound calls to communicate environmental states with conspecifics [1,2]. Over the last two decades, studies have shown that different categories of ultrasonic vocalizations emitted by rodents are associated with distinct environmental and motivational states. Specifically, 22–28 kHz ultrasonic vocalizations (USV)s are emitted in response to threatening and alarming situations [3,4,5], and 50–55 kHz frequency modulated (FM) USVs are emitted in response to rewarding stimuli [6,7,8]. Moreover, emission of these USV subtypes is dependent on distinct neurotransmitter pathways: the 22–28 kHz calls are produced by activation of the medial cholinoceptive vocalization strip [4,9,10] and 50–55 kHz FM USVs are generated through the activation of the ascending mesolimbic system [11,12,13,14,15,16]. Thus, spontaneous emissions of these USV subtypes (i.e., 22–28 kHz and 50–55 kHz FM calls) may serve as biomarkers of underlying neural activity in the cholinergic and dopaminergic system.

The dopaminergic and cholinergic systems also play an important role in the development of alcohol use disorders. For instance, alcohol is known to increase the efflux of dopamine into mesocorticolimbic regions [17,18], and administration of dopamine antagonists can reduce alcohol intake in rodents [19,20]. Moreover, variations in genes encoding catechol-o-methyltransferase (COMT), one of the enzymes responsible for the degradation of catecholamines, are associated with alcohol dependence [21,22,23]. Cholinergic receptors in the ventral tegmental area (VTA) have been shown to regulate the reinforcing properties of ethanol (EtOH) [24,25] and acute alcohol exposure can elevate muscarinic tone in the septohippocampal system [26]. Furthermore, cholinergic antagonist mecamylamine [27] and partial agonist varenicline [28] have been shown to reduce alcohol consumption behaviors. Variations in genes encoding nicotinic (CHRNA) and muscarinic (CHRM2) cholinergic receptors have also been associated with subjective responses to alcohol and the risk for alcohol dependence [29,30,31,32,33]. Together these results suggest that interindividual variation in alcohol consumption levels may in part be modulated by basal activity in the cholinergic and dopaminergic system.

Since USVs are reflective of activity in these systems, it is possible that USV transmission may be sensitive to modulation by EtOH and specific USV patterns may be associated with alcohol consumption behaviors in rodents. Indeed, recent work from our lab has shown that acute and chronic alcohol exposure can alter the counts and acoustic characteristics of spontaneously emitted USVs in alcohol-preferring (P) and high-alcohol-drinking (HAD-1) rats [34,35]. We have also shown that spontaneous USVs emitted in an alcohol-naïve state can be used to discriminate between HAD-1 and low-alcohol-drinking (LAD-1), as well as, P and alcohol non-preferring (NP) rats with a high degree of accuracy [36,37]. Furthermore, we have found that spontaneous USVs emitted by alcohol-naïve Long–Evans (LE) rats can be used to predict future alcohol consumption levels [38].

In the present study we extend our previous findings to explore the role of EtOH modulation on spontaneous USVs emitted by male LE rats. USVs were recorded from adult male LE rats for 4 h per day, 3 days per week, over the course of 24 weeks, under a variety of EtOH availability conditions. We used multivariate regression and linear mixed models to assess the effect of time and EtOH modulation on total counts and acoustic characteristics of 50–55 kHz FM and 22–28 kHz USVs. Based on previous work in our lab and the published literature, we hypothesized that USV counts and characteristics would be altered over time as the rats matured, and that these changes would be differentially modulated by EtOH exposure.

## 2. Materials and Methods

### 2.1. Animals

Twenty-four male Long–Evans rats were ordered from Harlan (Harlan Laboratories, Indianapolis, Indiana) at approximately 4 weeks of age. Rats were handled 5 days per week for 3 weeks to habituate them to the experimenters before the beginning of any experiments. All experiments were conducted in accordance with Institutional Animal Care and Use Committee guidelines.

The experimental procedures utilized for data collection were approved (4.2017) by the University of Texas Institutional Animal Care and Use Committee (IACUC). Protocol I.D. AUP-2017.0073.

### 2.2. Ultrasonic Vocalization Recordings

**Figure 1:**
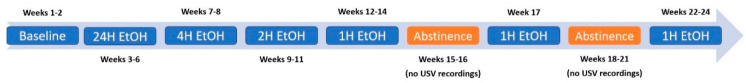
Schematic of experimental timeline. Ultrasonic vocalizations (USV)s were recorded from adult, male Long–Evans rats with variable access to 3-bottle choice ethanol (EtOH) (water, 15% EtOH, 30% EtOH) or water controls over a 24-week period.

USV recordings were conducted at the beginning of the dark cycle as described in previous studies [36]. All rats were recorded under both alcohol-naïve and alcohol-available (or water-available for control rats) conditions. Following the habituation period, USVs were recorded for 4 h and 10 min per day, 3 days/week over a 24-week experimental timeline (Figure 1). CM16 microphones were used with an UltraSound Gate interface (Avisoft Bioacoustics, Berlin, Germany) to record USVs at a 250-kHz sampling rate and a 16-bit resolution. On recording days, animals were weighed at the beginning of the dark cycle, transported to a test room, and placed into recording cages (which were identical to their home cage but only used for USV recordings). Recordings began immediately after the animals were placed in their respective cages. Following a 10-min “anticipatory” period, EtOH (or water) and food were introduced to the recording cages and USVs were recorded continuously for the next 4 h. Each animal was assigned its own recording cage in order to prevent any non-specific behaviors related to novelty or conspecific scents [39]. Based on rat and chamber size, we approximate the distance between the animal’s head and the centered microphone to range from 5 cm to 28.4 cm. After the recording session, the animals were transported back to the vivarium and returned to their home cage.

### 2.3. EtOH Consumption Timeline

At the beginning of the dark cycle, animals were weighed and then transported from the vivarium to the behavioral testing room. Animals were pair-housed during the habituation period, but were separated and single-housed before beginning the experimental sessions. The experimental sessions were conducted in the dark with only red illumination 3–5 days per week (3 days/week Weeks 1–12; 5 days/week Week 13–24) and commenced for a total of 24 weeks. Figure 1 provides a schematic of the experimental timeline employed in this study. During the first two weeks (Baseline), all rats had access to three sipper tubes filled with water only. Following the baseline phase, the rats underwent a chronic intermittent EtOH access paradigm with EtOH access three days per week as previously described [36,37,38]. For the next phase (24-h EtOH Access; 3 days/week; 4 weeks) the EtOH group had access to 3 sipper tubes (water, 15% and 30% EtOH) while the Control group had access to three water sipper tubes every other day (i.e., Monday, Wednesday, Friday). During this phase, animals had EtOH access during the entire 4-h USV recording session and then received continued EtOH access in their home cage for the remaining 20-h. For the following phase (4-h EtOH Access; 3 days/week; 2 weeks) animals in the EtOH group had 4-h access to EtOH every other day (i.e., Monday, Wednesday, Friday). During the next phase (2-h EtOH Access; 3 days/week; 3 weeks) animals in the EtOH group had 2-h access to EtOH every other day (i.e., Monday, Wednesday, Friday). After 2 h the EtOH bottles were replaced with three bottles of water and USV recordings continued for 2 more hours. For the next phase (1-h EtOH Access; 5 days/week; 3 weeks), the animals received 1-h access to EtOH 5 days per week (i.e., Monday–Friday), with USV recording sessions 3 days/week as in previous weeks (i.e., Monday, Wednesday, Friday). During the recording sessions, EtOH bottles were replaced with water bottles after 1 h and the recordings continued for 3 additional hours. Following the 1-h sessions, the animals underwent 2 weeks of “abstinence” where water was available, but not EtOH. USV recording sessions were not conducted during the abstinence period. After the 2-week abstinence period, animals underwent a 1-week “reinstatement” period which was similar to the 1-h EtOH access period (i.e., 1-h EtOH access 5 days/week, USV recordings 3 days/week). Following the first reinstatement period the animals underwent a second abstinence period for 4 weeks. As before, water, but not EtOH was available during this period. USVs were recorded during this final Abstinence phase. For the final phase of the experiment, the animals underwent a second reinstatement period for 3 weeks, which was similar to the 1-h EtOH access (i.e., 1-h EtOH access 5 days/week, USV recordings 3 days/week) and the first reinstatement period. Fluid intake was assessed gravimetrically after each drinking interval.

### 2.4. WAAVES Automated Analysis of USVs

Ultrasonic vocalization recordings were analyzed using the WAAVES program [40,41]. This program reads audio files and produces a frequency spectrogram. The spectrogram is then scanned for sound objects using MATLAB’s Image Processing Toolbox (MathWorks, Inc. Natick, MA, USA). For 50–55 kHz FM USVs, WAAVES identifies sound objects with a minimum duration of 5 ms occurring in a range of 30–120 kHz. An inter-call interval of 10 ms was used to discriminate between individual calls and avoid counting call fragments as separate calls. FM USVs were defined as calls that varied more than 5 kHz over the entire duration of the call. The 22–28 kHz calls were identified as sound objects occurring in a frequency range of 20 to 30 kHz with a minimum duration of 200 ms. An inter-call interval of 100 ms was used to separate individual calls. These call parameters were derived from the existing literature as well as extensive trial-and-error testing in the laboratory. Once the calls were identified, several measurements of interest were extracted from each USV call and stored for subsequent analysis. The mean frequency, duration, bandwidth, and power for both 50–55 kHz FM and 22–28 kHz calls were used for statistical analysis.

### 2.5. Validation Process for WAAVES Automation

WAAVES-generated USV data were validated through correspondence with human-derived (hand-scored) analyses. A subset of 10-min USV files recorded from LE rats (22–28 kHz calls: 40 files; 50–55 kHz calls: 40 files) were used for manual validation. The total number of calls identified via manual analysis was correlated with the total number of calls identified by the automated WAAVES program. The correlation coefficients are reported in the results.

### 2.6. Statistical Analyses of USV Counts and Acoustic Characteristics

Linear mixed models were used to analyze USV counts and acoustic characteristics as previously described [36,37]. We have found that this method provides a richer view of USV data than a standard statistical approach utilizing repeated measures ANOVA. For instance, using ANOVA, all calls emitted by a rat are used to calculate an average, and then any potential group differences in these averages are assessed. Thus, this standard method results in loss of important information about the inter-individual variability in USV calls for each rat, which, in turn, reduces power. Linear mixed models allow us to overcome these problems by accounting for inter-individual variation while examining the effects of treatment (e.g., EtOH vs. Control) and experiment stage on total USV counts and the patterns of USV acoustic characteristics (e.g., mean frequency, duration, bandwidth, or power).

#### 2.6.1. Linear Mixed Models

We assessed differences in total USV counts and each of the four USV characteristics as a function of treatment or experimental stage using a linear mixed model (LMM) in R (R Core Team, 2015) using the package “lmerTest” [42]. The linear models were generated to assess the effect of treatment, experimental phase, or an interaction of these factors for USV counts and each of the 4 acoustic characteristics of interest. Whenever a significant effect was observed a new reduced model was generated by removing the significant factor and compared with the original model using an ANOVA in order to assess the impact of the respective factor on the goodness-of-fit for the model. A random slope coefficient was included to protect against potential noise introduced by random day-to-day variation in call parameters for each rat.

#### 2.6.2. Linear Regression

We used linear regression modeling to assess the effect of rat age on USV counts and acoustic characteristics. Since all subjects were age matched at the beginning of the experiment, the week of recording was used as a proxy for age.

## 3. Results

The goal of this study was to investigate the effects of age and EtOH exposure on the counts and acoustic characteristics of spontaneously emitted 50–55 kHz FM and 22–28 kHz USVs in adult male Long–Evans rats. USVs were recorded 3 days/week, over 24 weeks under control (water only) or EtOH conditions (Figure 1). The average EtOH consumption per rat during each experiment stage was as follows—4 H (Weeks 3–6): 1.0 ± 0.06 g/kg/rat; 24 H (Weeks 3–6): 3.2 ± 0.2 g/kg/rat; 4 H (Weeks 7–8) 1.3 ± 0.3 g/kg/rat; 2 H (Weeks 9–11): 0.75 ± 0.05 g/kg/rat; 1 H (Weeks 12–14): 0.72 ± 0.05 g/kg/rat; Reinstatement 1 (Week 17): 0.68 ± 0.06 g/kg/rat; Reinstatement 2 (Weeks 22–24): 0.55 ± 0.04 g/kg/rat. USV recording sessions consisted of a 10-min anticipatory period during which animals were placed in the recording chambers and recorded in the absence of food and water (or EtOH). Following the anticipatory period food and water (or EtOH) were added to the recording cages and USVs were continuously recorded for 4 h.

### 3.1. Rate of USV Emissions

**Figure 2:**
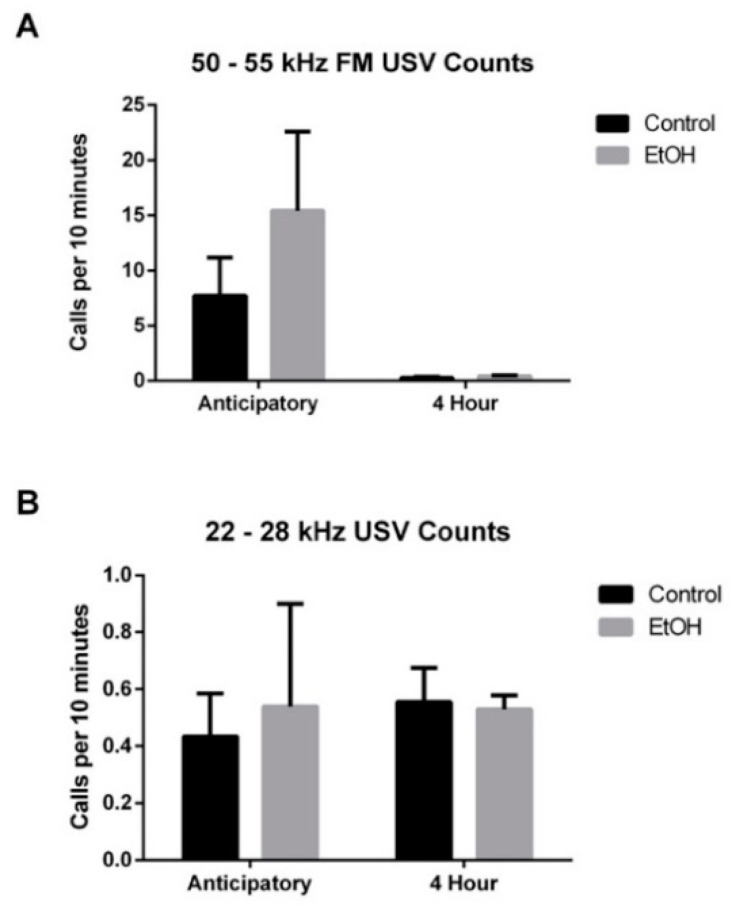
Rate of USV emissions during 10-min anticipatory and subsequent 4-h access periods. (**A**) The rate of 50–55 kHz frequency modulated (FM) USVs emitted during the anticipatory period was significantly higher compared to the subsequent 4-h session (*p* < 0.05). However, there was no difference in this rate between EtOH and control rats. (**B**) There was no difference in the rate of 22–28 kHz USV emissions between the anticipatory or the 4-h periods, nor between EtOH or water treated rats.

We began by examining the rate of 50–55 kHz FM and 22–28 kHz USVs emitted during the entire 24-week experiment. Since the anticipatory period is 10-min in duration, USV emission rate was measured in 10-min bins. We found that the rate of calling for 50–55 kHz FM USVs emitted during the anticipatory period (Control: 7.715 ± 3.466; EtOH: 15.415 ± 7.181) was significantly higher than those emitted during the subsequent 4-h recording session (Control: 0.291 ± 0.065; EtOH: 0.427 ± 0.090; *F*_1,22_ = 4.616, *p* < 0.05; Figure 2A). Moreover, while both EtOH and Control treated animals had a higher rate of 50–55 kHz FM USV emissions during the anticipatory period, only the EtOH treated animals showed a significant difference in the rate of emission (*t*_22_ = 2.489, *p* < 0.05; Sidak correction for multiple comparisons). Within the EtOH treated group we did not observe any significant correlation between EtOH intake and 50–55 kHz FM USV emissions during either the anticipatory (*R*^2^ = 0.04) or the 4-h recording session (*R*^2^ = 0.02). On the other hand, the 22–28 kHz USV emission rate was comparable between the anticipatory period (Control: 0.433 ± 0.151; EtOH: 0.576 ± 0.382) and the subsequent 4-h recording session (Control: 0.555 ± 0.119; EtOH: 0.530 ± 0.048; Figure 2B). Moreover, we did not observe any significant correlation between EtOH intake and 22–28 kHz USV emissions during either the anticipatory (*R*^2^ = 0.03) or the 4-h recording session (*R*^2^ = 0.10).

### 3.2. Effect of Age on USV Emissions

Since the animals were approximately 2 months old at the time of the first recording session and approximately 8 months old by the time of the final recording session, we next sought to examine the effect of age on USV acoustic counts and characteristics. Since we were only interested in the effect of age, we focused solely on the water treated control animals for this analysis. We used linear regression to determine whether USV counts and acoustic characteristics changed as a function of time. USVs emitted during the anticipatory and the 4-h recording periods were analyzed separately.

#### 3.2.1. 50–55 kHz FM USVs

**Figure 3:**
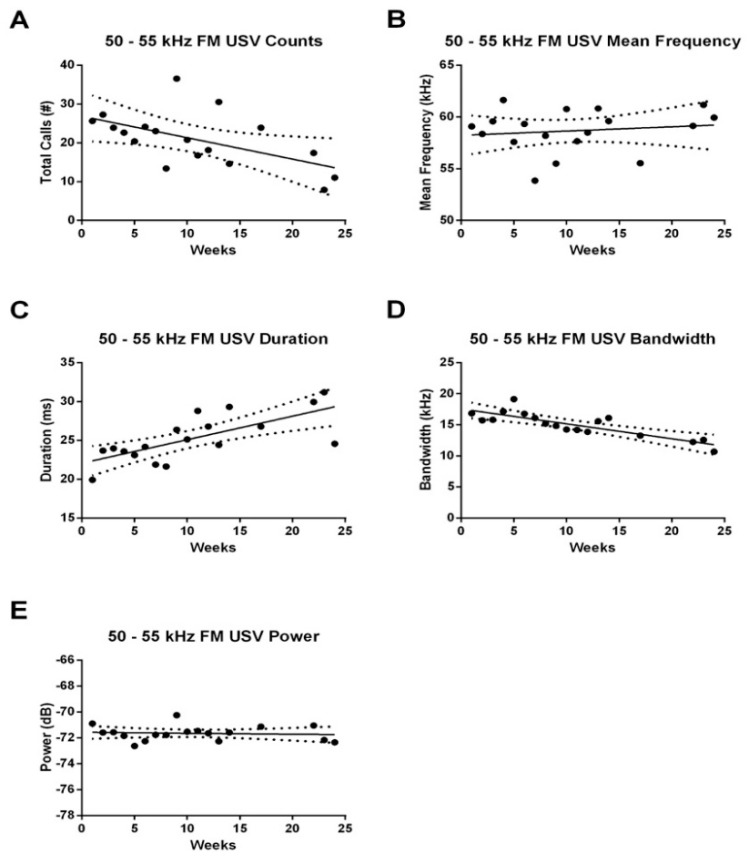
Correlation between age and 50–55 kHz FM USV counts and acoustic characteristics during the 4-h recording period. (**A**) There was a significant reduction in total 50–55 kHz FM USV counts as the animals aged (*p* < 0.05). (**B**) Mean frequency of 50–55 kHz FM USVs did not vary with age. There was an increase in (**C**) duration, and decrease in (**D**) bandwidth with age. (**E**) Power did not vary with age. Dots represent the group average across all control rats during each of the recording weeks.

We did not observe any significant relationship between time and weekly counts or acoustic characteristics of 50–55 kHz FM USVs emitted during the anticipatory period. On the other hand, we saw a significant reduction in 50–55 kHz FM USV emissions over time during the 4-h recording period (*F*_1,142_ = 4.660, *p* < 0.05; *r*-squared = 0.0318; Figure 3A). Moreover, there was also an increase in the duration (*F*_1,141_ = 13.440, *p* < 0.001; *r*-squared = 0.0870; Figure 3C) and a decrease in the bandwidth (*F*_1,141_ = 19.440, *p* < 0.0001; *r*-squared = 0.1210; Figure 3D) of these calls over time. No such effect of time was observed on the mean frequency (Figure 3B) or power (Figure 3E) of these calls.

#### 3.2.2. 22–28 kHz FM USVs

**Figure 4:**
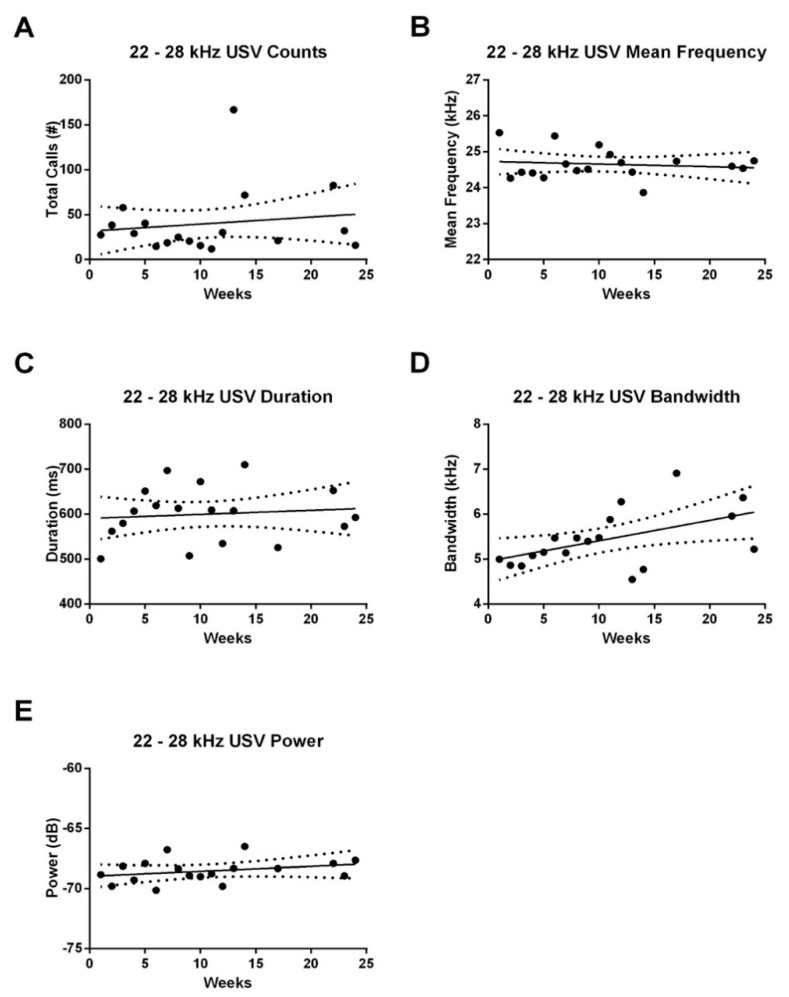
Correlation between age and 22–28 kHz USV counts and acoustic characteristics during the 4-h recording period. (**A**) There was no change in total 22–28 kHz USV counts as the animals aged. (**B**) Mean frequency or (**C**) duration of 22–28 kHz USVs did not vary with age. (**D**) There was an increase in bandwidth with age. (**E**) Power of these calls did not vary with age. Dots represent the group average across all control rats during each of the recording weeks.

In line with the 50–55 kHz FM call data, we did not observe any significant relationship between time and weekly counts or acoustic characteristics of 22–28 kHz USVs emitted during the anticipatory period. Moreover, we also did not see any change over time in the total number of 22–28 kHz USVs emitted during the 4-h recording period (Figure 4A). However, there was a significant increase in the bandwidth of these calls over time (*F*_1,141_ = 5.185, *p* < 0.05; *r*-squared = 0.0355; Figure 4D). No such effect was seen in the mean frequency (Figure 4B), duration (Figure 4C) or power (Figure 4E) of these calls.

### 3.3. EtOH Modulation of USV Emissions

Finally, we used linear mixed models to assess the effect of EtOH treatment on the counts and acoustic characteristics of spontaneously emitted 50–55 kHz FM and 22–28 kHz USVs. The calls emitted during the anticipatory period were analyzed separately from those emitted during the 4-h recording session in the presence of water or EtOH.

#### 3.3.1. 50–55 kHz FM USVs

**Figure 5:**
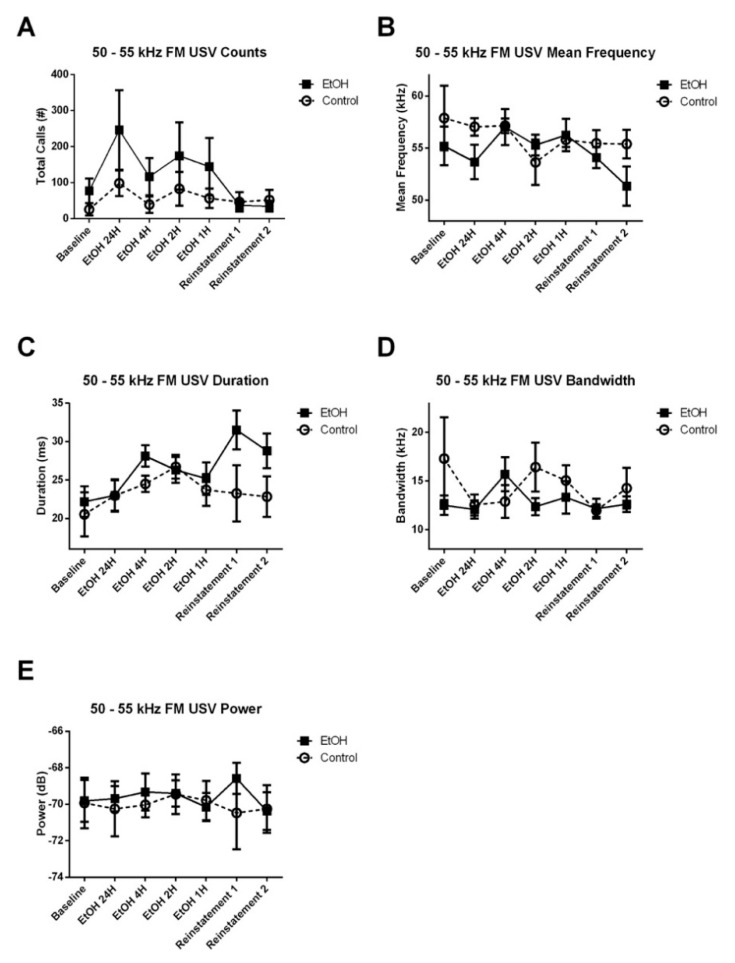
Effect of EtOH exposure on the anticipatory 50–55 kHz FM USV counts and acoustic characteristics. (**A**) There was no Treatment × Experiment Stage interaction, nor any main effect of treatment on the number of 50–55 kHz FM calls. (**B**) There was a significant Treatment × Experiment Stage interaction for mean frequency (*p* < 0.0001). There was no Treatment × Experiment Stage interaction, nor any main effect of treatment on the (**C**) duration or (**D**) bandwidth of these calls. (**E**) There was a significant Treatment × Experiment Stage interaction for the power of these calls (*p* < 0.001).

We did not observe any significant Treatment × Experiment Stage interaction, nor any main effect of treatment on the number of 50–55 kHz FM calls emitted during the anticipatory period (Figure 5A). However, there was a significant Treatment × Experiment Stage interaction for the mean frequency (Figure 5B) and power (Figure 5E), but not the duration (Figure 5C) or bandwidth (Figure 5D) of these calls. The significant interaction for mean frequency was observed in the linear (*t*_73_ = 2.225, *p* < 0.05) and the quadratic (*t*_1041_ = 4.818, *p* < 0.0001) terms of the model. Removal of this interaction resulted in a significant reduction in the goodness-of-fit of the model (*χ*^2^ = 28.323, *p* < 0.0001). The interaction for power was observed in the quadratic (*t*_936_ = −3.318, *p* < 0.001), cubic (*t*_6414_ = 2.144, *p* < 0.05) and the sixth order (*t*_16424_ = 2.931, *p* < 0.01) terms of the model. Removal of this interaction resulted in a significant reduction in the goodness-of-fit of the model (*χ*^2^ = 24.261, *p* < 0.001).

**Figure 6:**
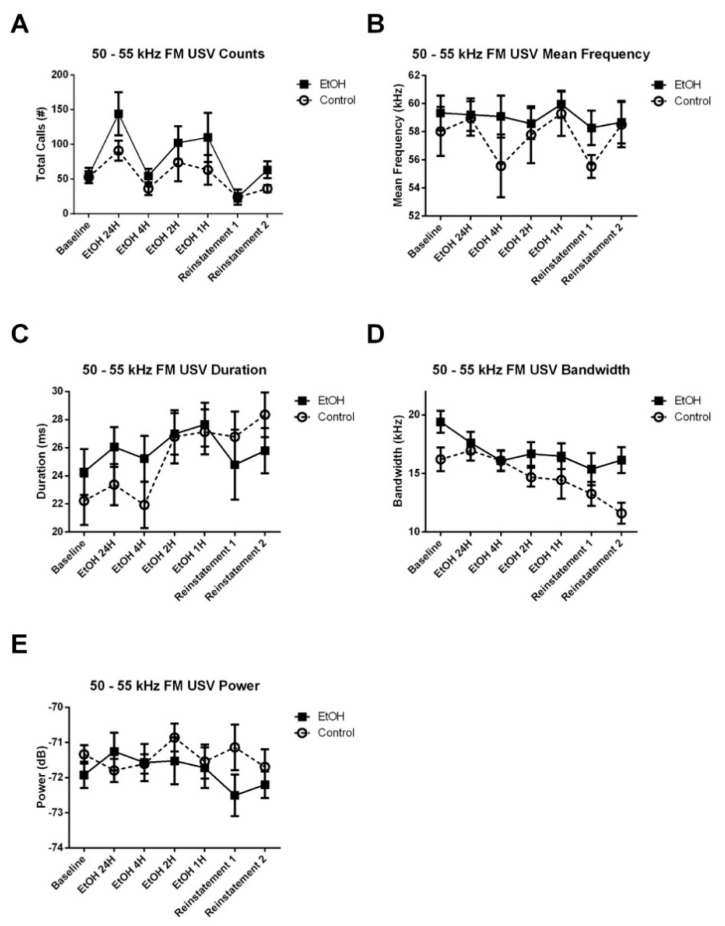
Effect of EtOH exposure on counts and acoustic characteristics of 50–55 kHz FM USVs emitted during the 4-h session. (**A**) There was no Treatment × Experiment Stage interaction, nor any main effect of Table 50–55 kHz FM calls. (**B**) There was a significant Treatment × Experiment Stage interaction for mean frequency (*p* < 0.05) of these calls. (**C**) There was no Treatment × Experiment Stage interaction, nor any main effect of treatment on duration. There was a significant Treatment × Experiment Stage interaction for (**D**) bandwidth (*p* < 0.05), and (**E**) power (*p* < 0.05) of these calls.

Similar to the anticipatory period results, we did not observe any significant Treatment × Experiment Stage interaction, nor any main effect of treatment on the number of 50–55 kHz FM calls emitted during the 4-h recording period (Figure 6A). However, there was a significant Treatment × Experiment Stage interaction for the mean frequency (Figure 6B), bandwidth (Figure 6D) and power (Figure 6E), but not the duration (Figure 6C) of these calls. The significant interaction for mean frequency was observed in the cubic (*t*_7955_ = 2.051, *p* < 0.05) and the fifth order (*t*_11843_ = −2.936, *p* < 0.01) terms of the model. Removal of this interaction resulted in a significant reduction in the goodness-of-fit of the model (*χ*^2^ = 17.369, *p* < 0.01). The interaction for bandwidth was observed in the quadratic (*t*_4887_ = 3.236, *p* < 0.01) term of the model. Removal of this interaction resulted in a significant reduction in the goodness-of-fit of the model (*χ*^2^ = 15.553, *p* < 0.05). The interaction for power was observed in the quadratic (*t*_3132_ = −3.305, *p* < 0.001), and the fifth order (*t*_1184_ = 2.177, *p* < 0.05) terms of the model. Removal of this interaction resulted in a significant reduction in the goodness-of-fit of the model (*χ*^2^ = 26.419, *p* < 0.001). Post-hoc assessment of the effect of Treatment on USV acoustic characteristics at each experiment stage did not reveal any significant effects. These results may partially be due to the fact that the total number of spontaneous USVs emitted during the 4-h period was low (~6 calls per rat per hour).

#### 3.3.2. 22–28 kHz USVs

We did not observe any significant Treatment × Experiment Stage interaction, nor any main effect of treatment on the number or the acoustic characteristics of 22–28 kHz USVs emitted during the anticipatory period. Although it is important to note that the lack of any effects is not particularly surprising due to the short duration of the anticipatory period resulting in very few total 22–28 kHz calls per animal (Control: 20.37 ± 7.09; EtOH: 25.37 ± 16.86) over the course of the experiment.

**Figure 7:**
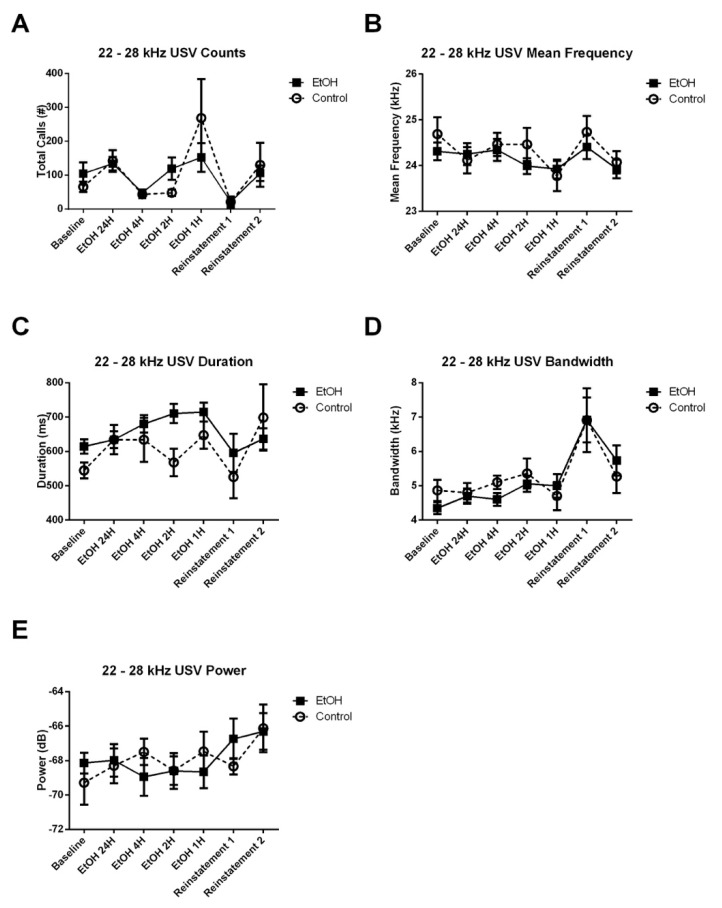
Effect of EtOH exposure on counts and acoustic characteristics of 22–28 kHz USVs emitted during the 4-h session. (**A**) There was no Treatment × Experiment Stage interaction, nor any main effect of treatment on the number of 22–28 kHz calls. (**B**) There was a significant Treatment × Experiment Stage interaction for mean frequency (*p* < 0.0001), (**C**) duration (*p* < 0.0001), (**D**) bandwidth (*p* < 0.001), and (**E**) power (*p* < 0.0001) of these calls.

Similar to the anticipatory period results, we did not observe any significant Treatment × Experiment Stage interaction, nor any main effect of treatment on the number of 22–28 kHz calls emitted during the 4-h recording period (Figure 7A). However, there was a significant Treatment × Experiment Stage interaction for the mean frequency (Figure 7B), duration (Figure 7C), bandwidth (Figure 7D) and power (Figure 7E) of these calls. The significant interaction for mean frequency was observed in the linear (*t*_30_ = 2.314, *p* < 0.05), the fourth order (*t*_16700_ = −3.439, *p* < 0.001) and the sixth order (*t*_16710_ = 4.160, *p* < 0.0001) terms of the model. Removal of this interaction resulted in a significant reduction in the goodness-of-fit of the model (*χ*^2^ = 76.167, *p* < 0.0001). The significant interaction for duration was observed in the quadratic (*t*_1140_ = −3.876, *p* < 0.001), cubic (*t*_9871_ = −9.498, *p* < 0.0001), fourth order (*t*_16528_ = −5.656, *p* < 0.0001) and the sixth order (*t*_16118_ = −3.632, *p* < 0.001) terms of the model. Removal of this interaction resulted in a significant reduction in the goodness-of-fit of the model (*χ*^2^ = 128.44, *p* < 0.0001). The interaction for bandwidth was observed in the linear (*t*_37_ = 2.764, *p* < 0.01), cubic (*t*_7163_ = −2.080, *p* < 0.05) and the fourth (*t*_1671_ = −3.914, *p* < 0.0001) terms of the model. Removal of this interaction resulted in a significant reduction in the goodness-of-fit of the model (*χ*^2^ = 27.261, *p* < 0.001). The interaction for power was observed in the cubic (*t*_7721_ = −4.043, *p* < 0.0001), fourth order (*t*_16704_ = −3.656, *p* < 0.0001), fifth order (*t*_16375_ = −4.256, *p* < 0.0001) and the sixth order (*t*_16724_ = −5.107, *p* < 0.0001) terms of the model. Removal of this interaction resulted in a significant reduction in the goodness-of-fit of the model (*χ*^2^ = 51.133, *p* < 0.0001). Post-hoc assessment of the effect of Treatment on USV acoustic characteristics at each experiment stage reveled significant differences in the duration of 22–28 kHz USVs during the 24 H EtOH (*χ*^2^ = 7.813, *p* < 0.01) access and 2 H EtOH (*χ*^2^ = 8.113, *p* < 0.01) access stages. No further effect of treatment was observed on any other characteristics during any other stage of the experiment. These results may partially be due to the fact that the total number of spontaneous USVs emitted during the 4-h period was low (~3 calls per rat per hour).

### 3.4. Validation of USV Automated Analysis

WAAVES-automated analysis and manual analysis were highly correlated for both the 22–28 kHz calls (*r* = 0.996) and 50–55 kHz calls *r* = 0.94).

## 4. Discussion

This study examined the effect of age and EtOH exposure on spontaneous 50–55 kHz FM and 22–28 kHz USV emissions. Over the course of 24 weeks, we recorded USVs from adult male Long–Evans rats under alcohol-naïve, 24-h EtOH access, 4-h EtOH access, 2-h EtOH access and 1-h EtOH access conditions. The recording sessions began when the subjects were 2 months old and continued until they were approximately 8 months old. Therefore, we first assessed how USV counts and characteristics change over time while rats increase in age and size. Next, we investigated the effects of EtOH exposure on USV transmission. From these experiments a number of interesting results emerged, which were consistent with our hypotheses that acoustic characteristics of USVs spontaneously emitted by male Long–Evans rats change as a function of age and that these changes are sensitive to EtOH exposure.

This study provided novel evidence that Long–Evans rats spontaneously emit 22–28 kHz USVs. Here we also showed that Long–Evans rats emit increased amounts of 50–55 kHz FM, but not 22–28 kHz, USVs when initially placed into the recording chamber before any alcohol, food or water is introduced. This result suggests that 50–55 kHz FM USVs may be associated with anticipatory and exploratory behavior in these rats. Moreover, although both control and EtOH treated animals exhibited increased calling during the 10-min “anticipatory” periods, only the EtOH group had a significantly higher emission rate during the anticipatory period than during the remaining 4-h session. This high rate may be due, in part, to an anticipation of EtOH and is consistent with previous work showing similar increases in 50–55 kHz FM USVs in anticipation of cocaine or alcohol self-administration [43,44,45].

Interestingly, although we did not see any age-dependent changes in these spontaneously emitted anticipatory calls, here we provide novel evidence of a decrease in the 50–55 kHz FM, but not 22–28 kHz, USV emissions as these rats mature. We can also show that as these rats become older the spontaneously emitted 50–55 kHz calls increase in duration but decrease in complexity as measured by the bandwidth of these calls. This decrease in bandwidth with age is in line with the previously published literature showing a reduction in the complexity of 50–55 kHz calls emitted by 32-month old geriatric rats [46]. Here we show that such a decline in call bandwidth begins as early as 6-months in male Long–Evans rats. On the other hand, the bandwidth of spontaneously emitted 22–28 kHz calls increased with age. It is interesting that these two USV subtypes, which represent opposing motivational and environmental states undergo opposing age-related changes. These results might suggest antagonistic changes in the neurotransmitter systems that underlie 22–28 kHz and 50–55 kHz FM calls. However, more direct experiments are needed to further explore this hypothesis.

Lastly, we used linear mixed models to examine the effect of EtOH treatment on the counts and acoustic characteristics of spontaneously emitted 50–55 kHz FM and 22–28 kHz USVs. Here we report that although EtOH exposure did not significantly alter the total number of spontaneous USVs emitted as compared to the control group, EtOH exposure differentially modulated the effect of age on the acoustic characteristics of both 50–55 kHz FM and 22–28 kHz USVs. Although the rats in our study only consumed moderate amounts of alcohol compared to what has been observed in other intermittent access studies [47], we still found significant EtOH treatment by time interactions on the mean frequency, bandwidth and power, but not duration, of 50–55 kHz FM USVs. We also observed significant EtOH treatment by time interactions on all four of these acoustic characteristics of spontaneously emitted 22–28 kHz USVs. Since we did not observe any clear differences in USV acoustic characteristics between the EtOH and control group during acute intoxication in the 24-h and 4-h access stages, we expect that the differences observed in this study may be due to the generalized effects of ethanol exposure vs. the water-only control group, rather than the acute effects of ethanol being in the system of the EtOH rats during the USV transmission. These results are not particularly surprising since acute and chronic EtOH exposure affects the activity of dopaminergic and cholinergic systems [30,31,48,49,50,51], which are known to underlie the transmission of 50–55 kHz FM and 22–28 kHz USVs [2,3,6,52,53]. These results suggest that USVs, in serving as a functional marker of dopaminergic and cholinergic activity, may provide useful information about the neurochemical changes produced by acute and chronic EtOH exposure.

## 5. Conclusions

In summary, this study adds to the breadth of existing literature highlighting the utility of USVs and USV acoustic characteristics as important non-invasive, behavioral correlates of the neurological changes induced by alcohol and other drugs of abuse. The USV characteristics assessed in the current study have been previously shown to differentiate between a variety of different behavioral and physiological phenomena, including discriminating between male and female rats, high alcohol drinking (HAD-1) and low alcohol drinking (LAD-1) rats, alcohol preferring (P) rats and non-alcohol preferring (NP) rats [54]. Furthermore, we have also shown that these characteristics can be used to predict future alcohol consumption behaviors in alcohol-naïve Long–Evans rats [38]. Although it is not yet well understood what each of the four USV acoustic parameters characterized in this study represent (i.e., mean frequency, duration, bandwidth, and power), changes in these parameters are known to represent changes in the underlying neural pathways in the cholinergic and the dopaminergic systems. Future research should explore the specific mappings between each USV acoustic characteristic and the corresponding neural transmission.
